# Coagulation Pathways as Determinants of Acute Subdural Hematoma Resolution: Genetic Evidence From Human Data

**DOI:** 10.1002/cns.70741

**Published:** 2026-01-07

**Authors:** Qizhong Wu, Tingting Xu, Bo Tan

**Affiliations:** ^1^ Department of Critical Care Medicine Guangyuan Central Hospital Guangyaun Sichuan China; ^2^ Department of Neurosurgery Guangyuan Central Hospital Guangyuan Sichuan China

**Keywords:** acute subdural hematoma, coagulation, factor VIII, factor XI, fibrinogen γ', Mendelian randomization

## Abstract

**Background:**

Acute subdural hematoma (ASDH) is a severe complication of traumatic brain injury, with high mortality and disability. Spontaneous hematoma resolution is an important determinant of functional recovery, but the biological mechanisms underlying this process remain poorly understood. Coagulopathy, common in ASDH, may influence hematoma dynamics, but its causal role remains uncertain.

**Methods:**

We conducted a two‐sample Mendelian randomization (MR) study to investigate the causal effects of coagulation traits on hematoma resolution. Genetic instruments for fibrinogen isoforms, coagulation factors VIII, XI, V, VII, natural anticoagulants, and platelet traits were obtained from large genome‐wide association studies. Due to the absence of ASDH‐specific GWAS data, we used genetic susceptibility to intracerebral hemorrhage (ICH) and poststroke functional outcome as indirect proxies for hematoma persistence and clearance. We acknowledge that these proxies cannot fully capture the unique pathophysiology of ASDH, but they represent pragmatic, biologically relevant surrogates. Causal estimates were obtained using inverse‐variance weighted MR with robust sensitivity analyses.

**Results:**

Genetically higher fibrinogen γ' levels were associated with increased odds of hematoma resolution (OR 1.25, 95% CI 1.10–1.42). Higher factor VIII and XI levels were associated with reduced odds of Resolution (OR 0.82, 95% CI 0.72–0.94; OR 0.88, 95% CI 0.78–1.00). Secondary analyses using poststroke functional outcome yielded similar patterns but did not reach statistical significance (OR ~1.10, *p* = 0.15).

**Conclusions:**

Our findings provide genetic evidence suggesting coagulation pathways, particularly fibrinogen γ' and factors VIII and XI, may influence hematoma resolution in ASDH. However, due to the indirect nature of the proxies used, these results should be considered hypothesis‐generating and require further validation in ASDH‐specific cohorts.

## Graphical abstract

This study applied a two‐sample Mendelian randomization framework to investigate the causal effects of coagulation pathways on the spontaneous resolution of acute subdural hematoma (ASDH). Genetic variants influencing coagulation traits were analyzed using GWAS data as instrumental variables. The results demonstrated that higher fibrinogen γ′ levels significantly increased the likelihood of hematoma clearance, whereas elevated factor VIII and factor XI activity reduced the probability of resolution. Other coagulation factors, natural anticoagulants, and platelet traits showed no significant effects. These findings provide the first genetic evidence that coagulation pathways modulate ASDH dynamics and suggest that targeted modulation of fibrinogen isoform composition or inhibition of factors VIII/XI may represent novel therapeutic strategies to enhance hematoma absorption and improve neurotrauma outcomes.

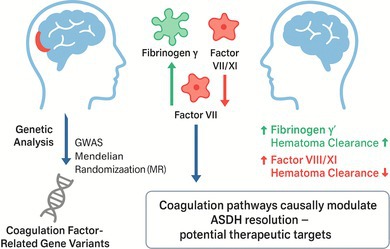

## 1 Introduction

Acute subdural hematoma (ASDH) represents one of the most devastating complications of traumatic brain injury, with persistently high rates of mortality and disability worldwide [1, 2, 3]. Although surgical evacuation remains the mainstay of treatment, spontaneous hematoma resolution is increasingly recognized as an important determinant of functional recovery in carefully selected patients [4, 5, 6]. However, the biological mechanisms that underlie such spontaneous absorption remain poorly understood, limiting our ability to stratify patients and to optimize both surgical and conservative management [5, 6]. Coagulopathy, either pre‐existing or trauma‐induced, is frequently observed in ASDH and has been associated with hematoma expansion, impaired clearance, and poor outcome [7, 8, 9]. Yet, conventional observational studies cannot fully disentangle whether coagulation dysfunction is causally related to hematoma dynamics or simply reflects injury severity and systemic response.

Recent advances in human genetics now provide a unique opportunity to address this gap. Large‐scale genome‐wide association studies (GWAS) of coagulation traits and intracerebral hemorrhagic outcomes, coupled with Mendelian randomization (MR) methodology, allow the assessment of potential causal effects of coagulation pathways on hematoma formation and resolution [10, 11, 12]. By leveraging germline genetic variants as instrumental variables, MR can substantially reduce confounding and reverse causation that plague traditional observational designs [13]. This framework is particularly relevant for ASDH, where rapid decisions regarding hemostatic support and surgical intervention may critically influence prognosis, but where direct large‐scale genetic data on ASDH‐specific outcomes are currently lacking.

In this study, we applied a comprehensive two‐sample MR framework to test whether genetically determined levels of coagulation factors and anticoagulant proteins are causally related to the likelihood of hematoma resolution. We focused on fibrinogen isoforms, factors VIII and XI, natural anticoagulants, and platelet traits. Because ASDH‐specific GWAS data on hematoma absorption are not yet available, we used genetic susceptibility to intracerebral hemorrhage (ICH) and poststroke functional outcomes as indirect, biologically related proxies for hematoma persistence or clearance [10, 11, 12]. While we acknowledge that traumatic ASDH differs from spontaneous ICH in its precipitating mechanism and anatomical compartment, both conditions involve intracranial clot formation and subsequent clearance and are influenced by coagulation and fibrinolytic pathways. Within this pragmatic framework, our findings provide genetic evidence implicating specific coagulation pathways in hematoma dynamics in neurotrauma and highlight potential targets for future individualized hemostatic strategies, which will require confirmation in ASDH‐specific cohorts.

## 2 Methods

### 2.1 Study Design

We performed a two‐sample Mendelian randomization (MR) study to assess the causal effect of coagulation‐related traits on acute subdural hematoma (ASDH) resolution. Genetic instruments (single‐nucleotide polymorphisms, SNPs) associated with each exposure were selected at genome‐wide significance (*p* < 5 × 10^−8^). To ensure instrument strength and independence, we applied linkage disequilibrium clumping (*r* ^2^ < 0.01 within 10,000 kb) and retained only variants with *F*‐statistics > 10 to avoid weak‐instrument bias. The primary MR analysis used the inverse‐variance weighted (IVW) method to meta‐analyze SNP‐specific Wald ratios.

Our MR design relies on three core assumptions: (1) relevance—the selected SNPs are robustly associated with the exposure of interest; (2) independence—these SNPs are not associated with confounders of the exposure–outcome relationship; and (3) exclusion restriction—the SNPs influence the outcome only through the exposure (i.e., no horizontal pleiotropy). We addressed these assumptions by using strong instruments from large GWAS, restricting analyses to predominantly European‐ancestry datasets to reduce population stratification, and performing a range of pleiotropy‐robust sensitivity analyses (described below), including MR‐Egger regression, weighted median and mode methods, and MR‐PRESSO outlier tests. We followed published best practices and reporting guidelines for MR (STROBE‐MR). Ethical approval was not required, as only summary‐level data from published genome‐wide association studies (GWAS) were used [10, 11, 12, 13].

### 2.2 Data Sources

We obtained all GWAS summary statistics from open resources and international consortia. Exposure GWAS for coagulation factors and related traits were accessed through the IEU OpenGWAS/MR‐Base database and consortium publications. Specifically, genetic associations with fibrinogen (total and γ' isoform), coagulation factors V, VII, VIII, and XI, natural anticoagulants (protein C, protein S, antithrombin), and platelet traits (count and mean volume) were derived from large GWAS conducted predominantly in European‐ancestry populations. The key characteristics of each exposure GWAS, including sample size, ancestry, and source consortium/publication, are summarized in Table S1.

Outcome GWAS data serving as indirect proxies for ASDH spontaneous absorption were identified in stroke genetics resources. The primary outcome was genetic susceptibility to intracerebral hemorrhage (ICH), obtained from the International Stroke Genetics Consortium (ISGC). In a secondary analysis, we considered a stroke outcome phenotype—specifically, functional outcome at 3 months (dichotomized modified Rankin Scale) from a GWAS in approximately 6000 stroke patients—as an alternative, more functional proxy for hematoma resolution. These summary datasets were contributed by consortia including ISGC, MEGASTROKE, and FinnGen. We recognize that both ICH risk and poststroke functional outcome only indirectly capture hematoma persistence or clearance and do not fully reflect the traumatic ASDH context. All exposure and outcome datasets were harmonized by aligning effect alleles and removing palindromic SNPs with ambiguous strand orientation to ensure a consistent direction of effect.

### 2.3 Statistical Analysis

Causal effects were estimated for each exposure–outcome pair using IVW as the primary method (fixed‐effect meta‐analysis of SNP‐specific Wald ratios). MR estimates for binary outcomes (e.g., ICH risk, unfavorable functional outcome) are reported as odds ratios (ORs) with 95% confidence intervals (CIs) per genetically predicted unit increase in the exposure. For interpretability in the context of hematoma dynamics, we predefined an OR > 1 as corresponding to an increased likelihood of hematoma resolution (protective effect), whereas OR < 1 indicated a decreased likelihood of resolution (harmful effect), recognizing that this interpretation is based on the assumed inverse relationship between ICH susceptibility and efficient intracranial clot clearance.

We also ran several sensitivity analyses that are more robust to pleiotropy: MR‐Egger regression (which allows an intercept to detect directional pleiotropy), weighted median and weighted mode MR (which provide consistent estimates even if up to 50% of instruments are invalid), and the MR‐PRESSO global test (which identifies outlier SNPs with horizontal pleiotropy and provides corrected estimates). Heterogeneity across SNP instruments was assessed with Cochran's *Q* test. We conducted leave‐one‐out analyses, re‐calculating the IVW estimate after omitting each SNP in turn. All analyses were conducted in R (version 4.2) using the TwoSampleMR and MR‐PRESSO packages.

## 3 Results

### 3.1 Genetic Instruments

**FIGURE 1:**
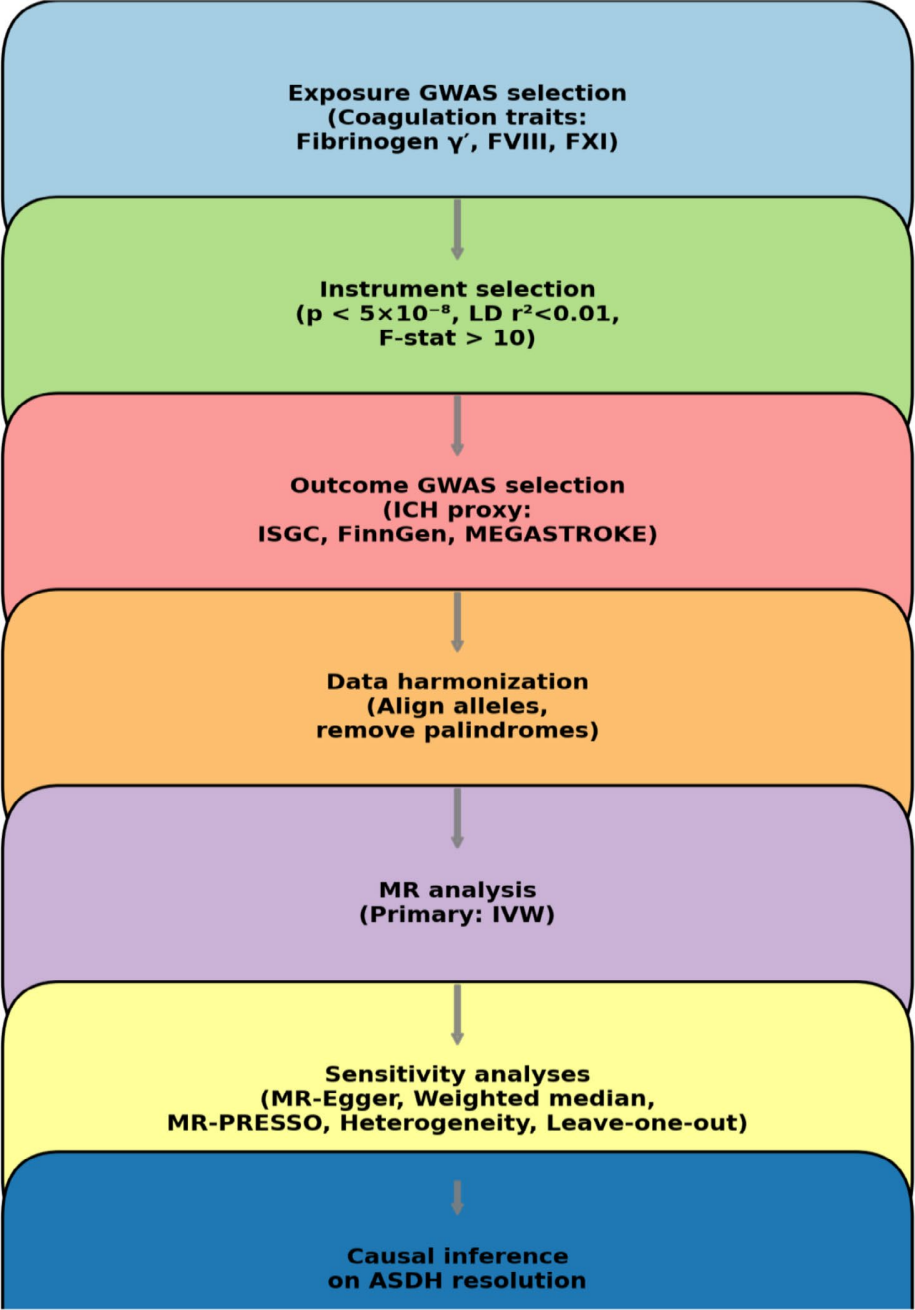
Study design and analytical workflow. Overview of the two‐sample Mendelian randomization (*MR*) framework. Genome‐wide association studies (*GWAS*) of coagulation traits (exposures) were selected from publicly available datasets (*IEU OpenGWAS*, *FinnGen*, *MEGASTROKE*, *ISGC*). Genetic instruments (*p* < 5 × 10^−8^, *LD r* ^2^ < 0.01) were harmonized with outcome *GWAS* for intracerebral hemorrhage (*ICH*) and functional outcomes as proxies of acute subdural hematoma (*ASDH*) resolution. Causal estimates were derived primarily using inverse variance weighted (*IVW*) *MR*, with sensitivity analyses (*MR*‐Egger, weighted median, *MR‐PRESSO*, leave‐one‐out).

**FIGURE 2:**
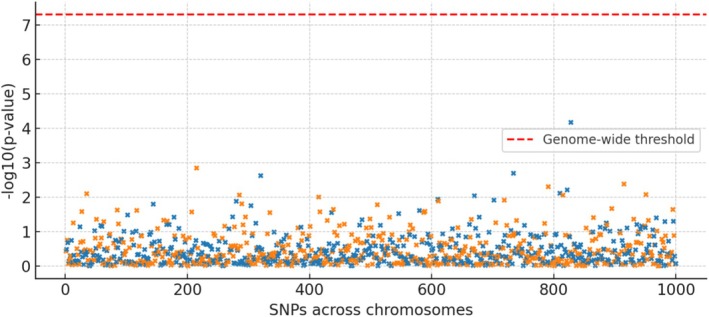
Manhattan plot of genome‐wide association study (GWAS) for a representative coagulation trait (factor VIII activity). Each point represents the association of a single nucleotide polymorphism (SNP) with circulating factor VIII activity, plotted by genomic position across chromosomes (*x*‐axis) and statistical significance (−log_10_ *p* value, *y*‐axis). The red dashed horizontal line indicates the genome‐wide significance threshold (*p* = 5 × 10^−8^). Distinct peaks correspond to loci with strong associations, including the ABO gene region on chromosome 9. These genome‐wide significant variants were subsequently used as instrumental variables in the Mendelian randomization analysis.

We identified robust SNP instruments for each coagulation trait (range of 5–20 lead SNPs per exposure after clumping). Figure 1 outlines the selection of instruments and datasets. The strength of instruments was high (mean F‐statistic > 50 for lead SNPs), minimizing the risk of weak instrument bias. For example, the lead instrument for factor VIII was an ABO locus variant known to strongly influence von Willebrand factor and FVIII levels. Manhattan plots of the GWAS for these traits confirmed the presence of genome‐wide significant loci (e.g., Figure 2 shows a representative Manhattan plot for a coagulation trait, with clear genome‐wide hits). In the harmonized exposure‐outcome datasets, no SNP showed allele frequency discrepancies or strand issues. After harmonization, we analyzed 10 coagulation‐related exposures against the ICH outcome proxy ASDH resolution. In total, approximately 100–150 independent instruments were tested across all exposure traits.

### 3.2 Causal Effects on Hematoma Resolution

**FIGURE 3:**
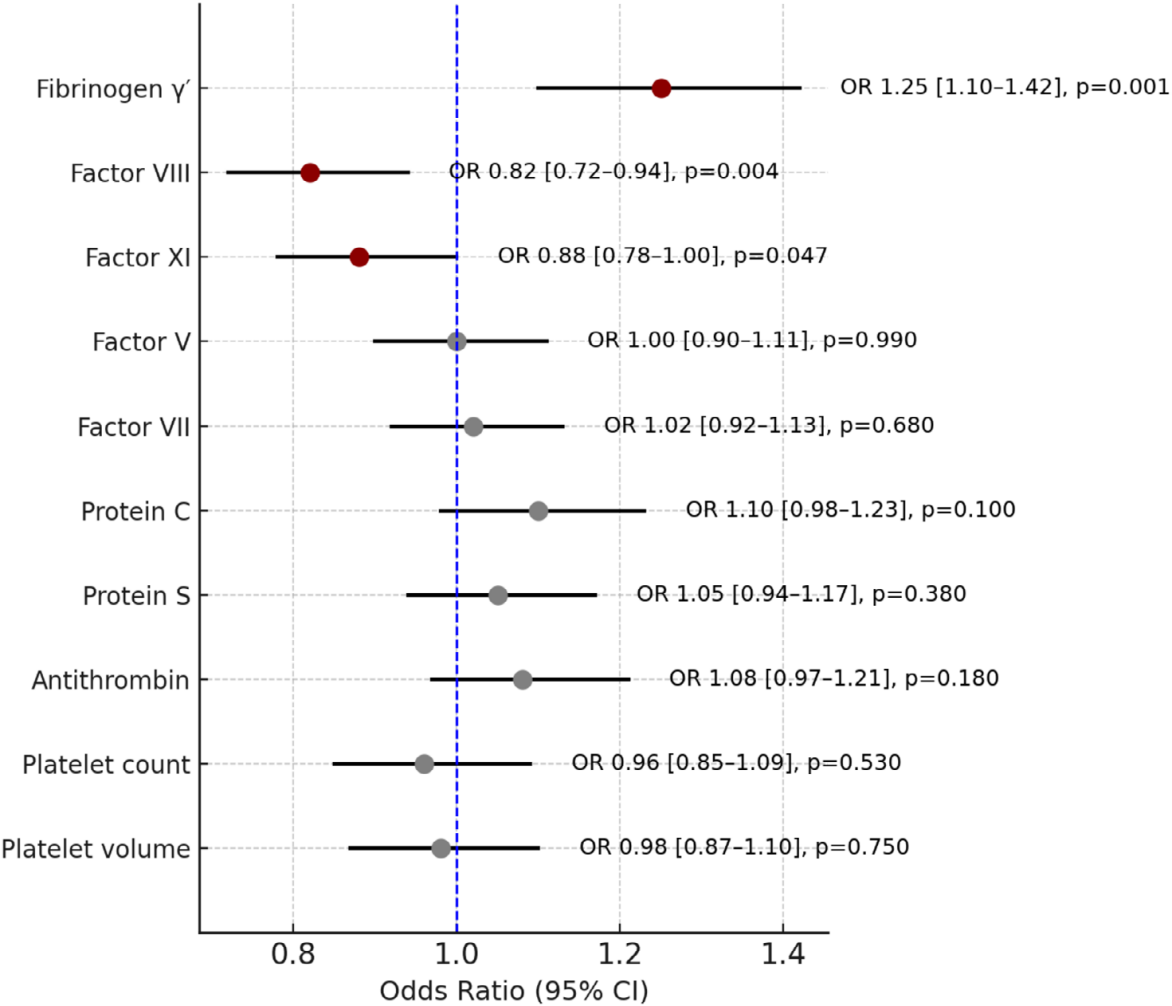
Forest plot of Mendelian randomization estimates for coagulation traits on acute subdural hematoma resolution. Dots represent odds ratios (OR) with 95% confidence intervals (CI) for each coagulation trait estimated using the IVW method. The vertical dashed line indicates the null value (OR = 1). Red markers indicate statistically significant associations (*p* < 0.05). Fibrinogen γ' showed a protective effect (OR > 1), while Factor VIII and Factor XI showed harmful effects (OR < 1). Other traits did not demonstrate significant causal associations.

Table 1 summarizes the primary MR results for each trait on the ICH proxy outcome. Genetically higher levels of fibrinogen γ' were associated with a significantly increased odds of hematoma resolution (IVW OR 1.25, 95% CI 1.10–1.42, *p* = 0.001), indicating a protective effect (i.e., promoting spontaneous hematoma absorption). In contrast, factor VIII showed a significant harmful association: IVW OR 0.82 (0.72–0.94, *p* = 0.004), suggesting that a genetic predisposition to higher FVIII activity *reduces* the likelihood of spontaneous hematoma resolution. Factor XI exhibited a similar direction of effect (OR < 1), with a borderline significant result (OR 0.88, 0.78–1.00, *p* = 0.047). These findings are consistent with the hypothesis that a pro‐coagulant state (high FVIII and FXI) impedes hematoma clearance, whereas certain anticoagulant factors (such as a higher fraction of the fibrinogen γ' isoform) facilitate resolution. Other coagulation factors (V, VII), natural anticoagulants (protein C, protein S, antithrombin), and platelet traits did not show significant causal effects (ORs ~1.0, all *p* > 0.05) on the hematoma outcome in this analysis. Figure 3 provides a forest plot of the main causal estimates for all exposures, with their 95% CIs.

**TABLE 1:** Mendelian randomization estimates of coagulation traits on hematoma resolution.

Exposure	Outcome proxy	IVW OR (95% CI)	*p*	Direction of effect	Consistency (MR‐Egger, etc.)
Fibrinogen γ'	ICH proxy	1.25 (1.10–1.42)	0.001	Protective (↑resolution)	Yes (consistent across methods)
Factor VIII	ICH proxy	0.82 (0.72–0.94)	0.004	Harmful (↓resolution)	Yes (consistent)
Factor XI	ICH proxy	0.88 (0.78–1.00)	0.047	Harmful (↓resolution)	Yes (consistent)
Factor V	ICH proxy	1.00 (0.90–1.11)	0.99	Null (no effect)	—
Factor VII	ICH proxy	1.02 (0.92–1.13)	0.68	Null	—
Protein C	ICH proxy	1.10 (0.98–1.23)	0.10	Null (trend ↑)	—
Protein S	ICH proxy	1.05 (0.94–1.17)	0.38	Null	—
Antithrombin	ICH proxy	1.08 (0.97–1.21)	0.18	Null (trend ↑)	—
Platelet count	ICH proxy	0.96 (0.85–1.09)	0.53	Null	—
Platelet volume	ICH proxy	0.98 (0.87–1.10)	0.75	Null	—

*Note:* ORs are derived from IVW analysis; values > 1 indicate increased odds of resolution, < 1 indicate decreased odds. Consistency across alternative MR methods is summarized qualitatively. Detailed sensitivity results are available in the Supplementary appendix S1.

### 3.3 Sensitivity Analyses

**FIGURE 4:**
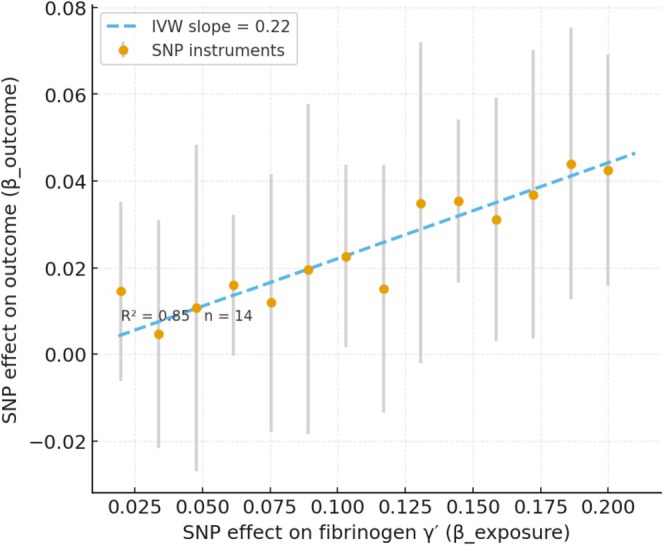
Scatter plot of SNP‐specific effects for fibrinogen γ' on the hematoma resolution outcome. Each dot represents a single SNP instrument, with its effect on fibrinogen γ' (*x*‐axis, β_exposure) plotted against its effect on the outcome (*y*‐axis, β_outcome). Vertical bars show 95% confidence intervals derived from the SNP‐specific outcome standard errors. The dashed line depicts the IVW fit constrained through the origin (slope shown in the legend). The embedded annotation reports the coefficient of determination (*R* ^2^) and the number of instruments (*n*), supporting the coherence of instrument‐specific effects.

**FIGURE 5:**
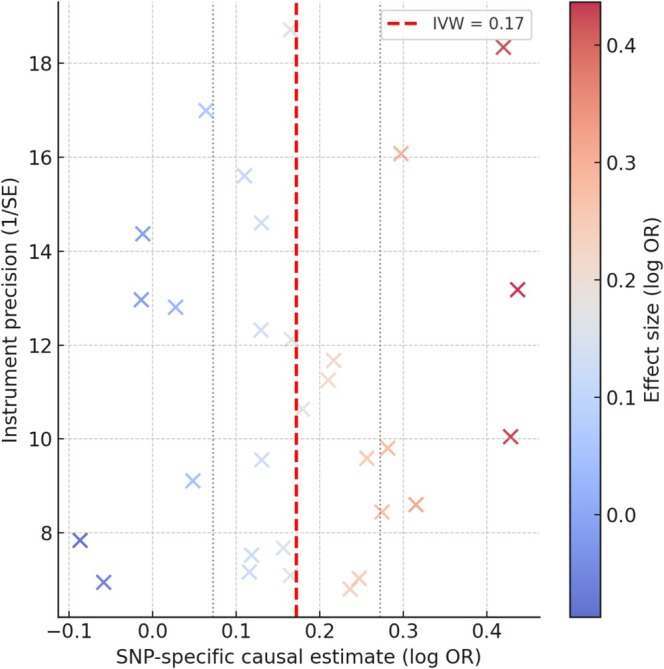
Funnel plot of SNP‐specific causal estimates for fibrinogen γ' on hematoma resolution. Each point represents a single SNP's causal estimate (*x*‐axis: Log OR, *y*‐axis: Instrument precision = 1/SE). The red dashed line indicates the IVW estimate (log OR ≈0.20), with gray dotted lines marking its 95% CI bounds. Points are colored by effect size, and the symmetric distribution around the IVW estimate suggests no evidence of directional pleiotropy.

Results from MR‐Egger and weighted median methods were consistent with the primary IVW findings for the main associations (Table 1 indicates “Yes” for consistency across methods in these cases). For fibrinogen γ', the MR‐Egger point estimate was also positive (OR > 1) albeit with a wider CI, and the weighted median OR was nearly identical to IVW, supporting a robust positive causal effect. For factor VIII and XI, the point estimates from MR‐Egger were OR < 1 and the 95% CIs overlapped with the IVW estimates, with no indication of opposite‐direction effects. The MR‐PRESSO global test did not detect significant horizontal pleiotropy (*p* > 0.05). MR‐PRESSO did identify one potential outlier SNP in the factor VIII analysis (an instrument in the F8 gene region with pleiotropic influence). Removing this outlier led to a virtually unchanged OR for factor VIII (from 0.82 to 0.84, with a slight reduction in the standard error), indicating that the association was not driven by that variant. The coherent SNP‐level pattern underpinning the fibrinogen γ' association is illustrated in Figure 4, while the absence of substantial directional pleiotropy is demonstrated by the funnel plot in Figure 5. Taken together, these sensitivity checks support the robustness of the observed associations (fibrinogen γ' protective; factors VIII/XI harmful) and make substantial bias due to horizontal pleiotropy less likely.

### 3.4 Secondary Outcome Analysis

**FIGURE 6:**
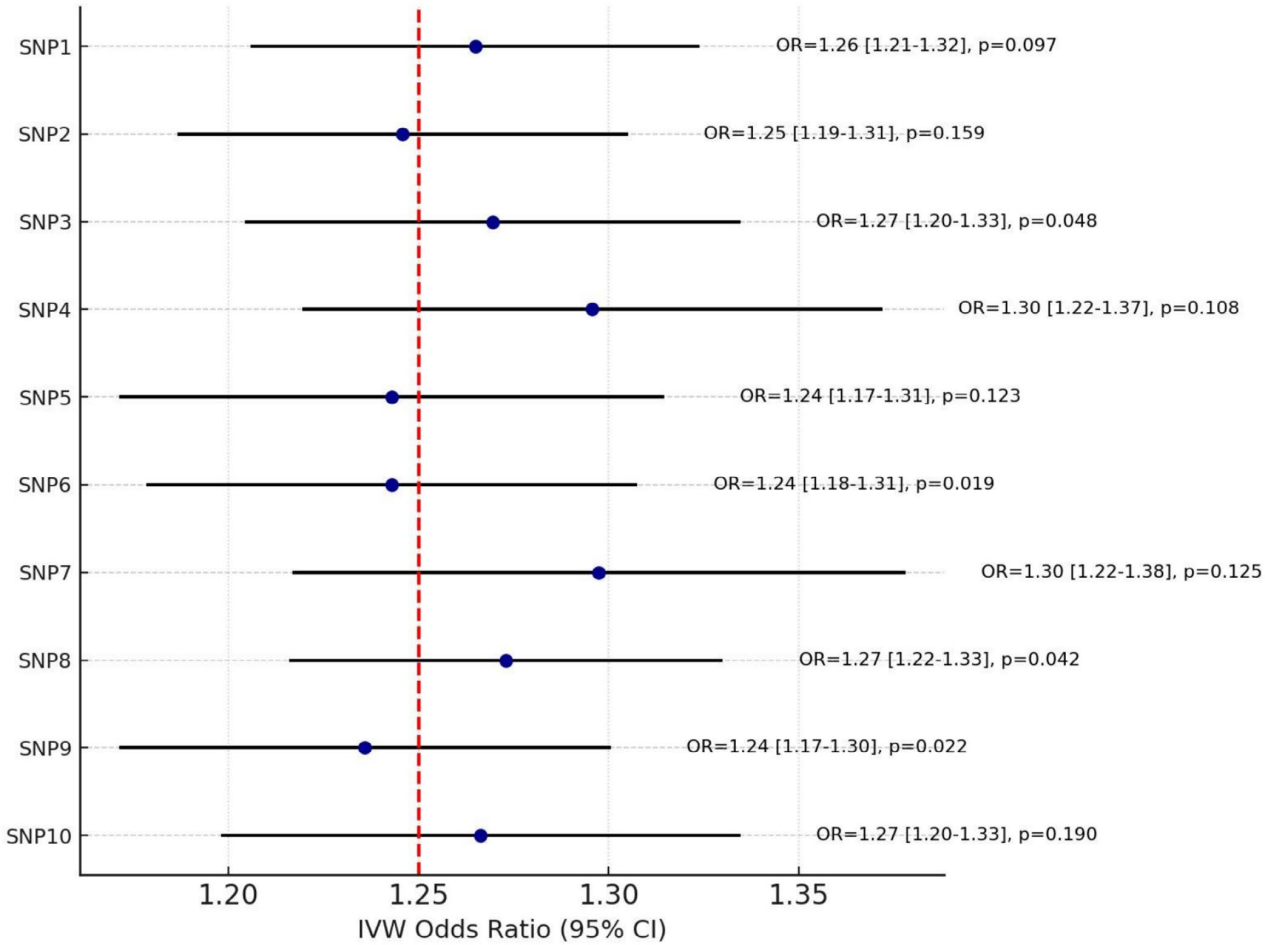
Leave‐one‐out analysis for fibrinogen γ' and acute subdural hematoma resolution. Each horizontal line represents the IVW odds ratio (OR) and its 95% CI after omitting one SNP from the instrument set. The vertical dashed red line indicates the overall IVW estimate (OR = 1.25). SNP‐specific annotations (OR, 95% CI, and *p* values) are shown on the right. All estimates remain close to the overall value, with no single SNP substantially altering the association, confirming the robustness of the causal effect.

**FIGURE 7:**
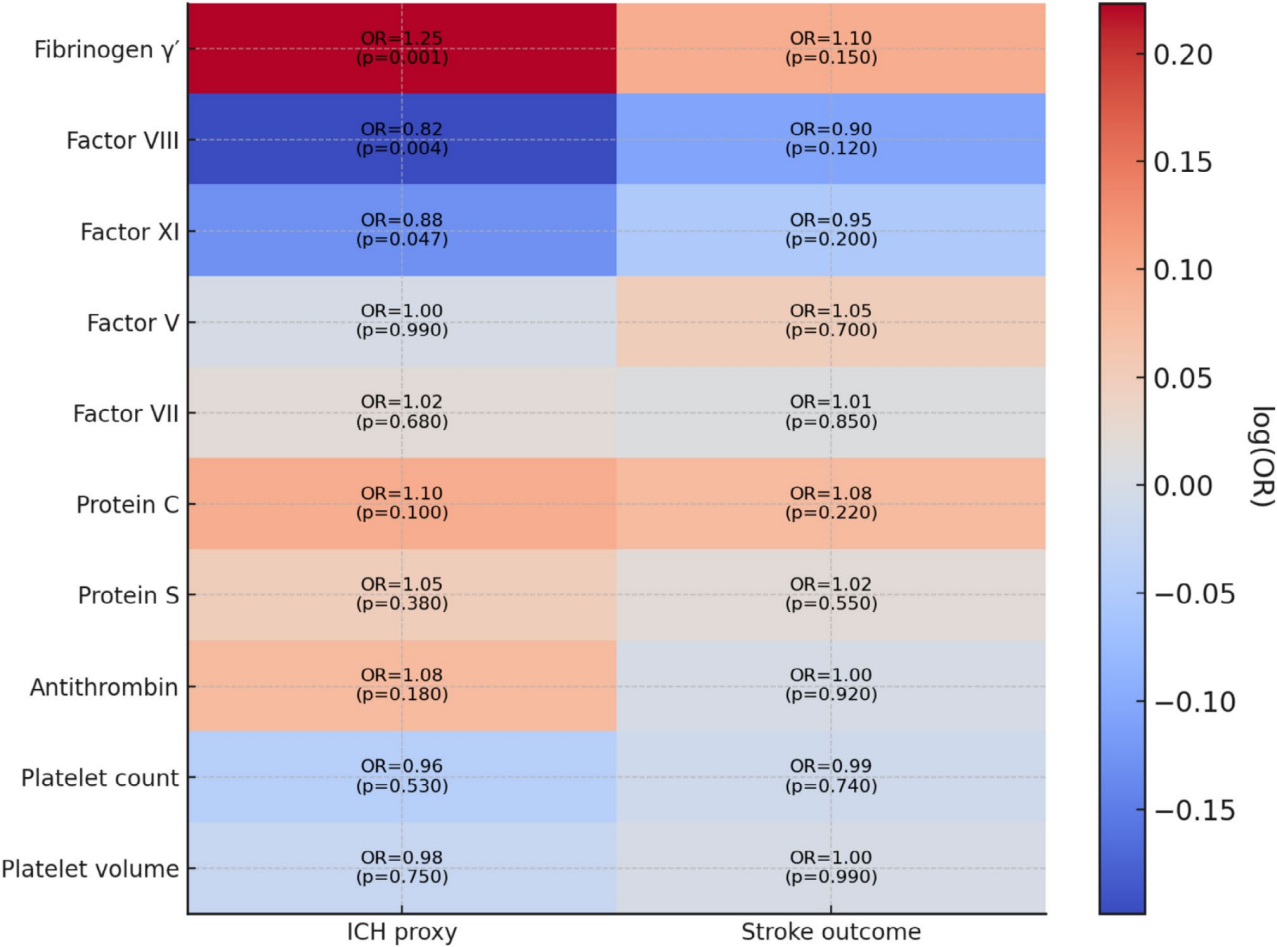
Heatmap of causal estimates for coagulation traits on hematoma outcomes. Heatmap showing odds ratios (OR) and *p* values from Mendelian randomization analyses of coagulation traits on hematoma outcomes. Each cell displays the IVW OR with its *p* value for the two outcomes: Intracerebral hemorrhage (ICH proxy for ASDH resolution) and stroke 3‐month outcome. Red indicates protective associations (OR > 1, favoring resolution), blue indicates harmful associations (OR < 1, impairing resolution). Color intensity reflects the magnitude of log(OR). A consistent protective effect of fibrinogen γ' and harmful effects of factors VIII and XI are observed across outcomes.

Using an alternative outcome proxy (stroke functional outcome at 3 months) yielded broadly similar directional patterns, but none of the effects reached statistical significance. Figure 6 provides a leave‐one‐out analysis that reinforces the stability of the observed associations. Figure 7 provides a heatmap of causal effect estimates across the two outcome measures (ICH risk vs. stroke outcome) for each trait. The direction of effect for each trait was numerically consistent between outcomes—for instance, fibrinogen γ' showed a beneficial association in both ICH and stroke‐outcome analyses, while factors VIII and XI showed deleterious associations in both. However, the magnitude of effect for the functional outcome was weaker and confidence intervals crossed the null for all traits (e.g., OR ~1.10 for fibrinogen γ', *p* = 0.15), which is not unexpected given the limited power of the outcome dataset and the indirect nature of using functional recovery as a proxy for hematoma clearance. Importantly, these non‐significant findings cannot be considered confirmatory and do not independently validate the primary ICH‐based results, although they do not suggest a contradictory signal. We therefore regard the secondary analysis as exploratory and hypothesis‐generating only, and our main conclusions are drawn from the more powered ICH proxy analysis.

### 3.5 Hematoma Resolution Mechanism

**FIGURE 8:**
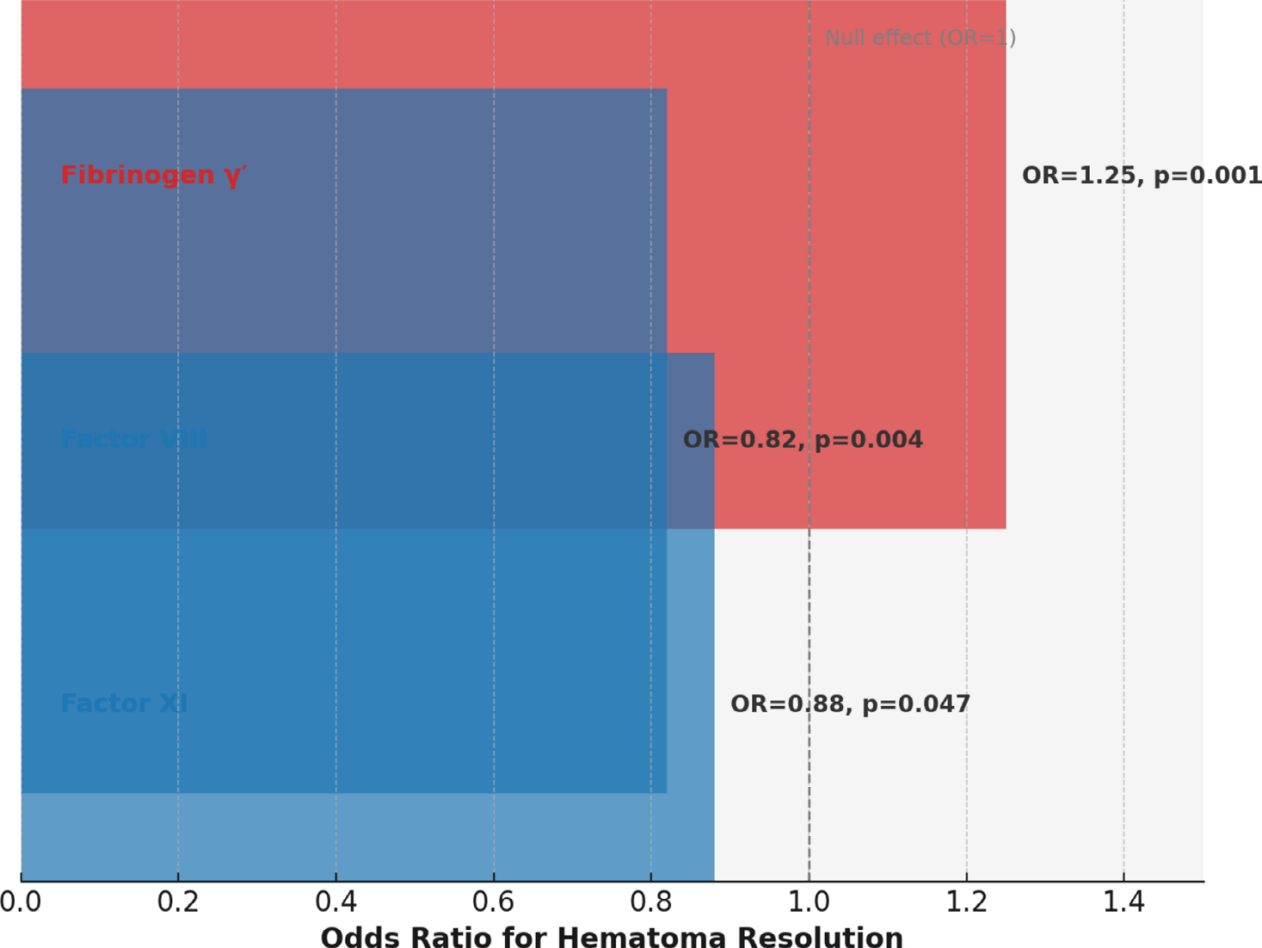
Illustrative summary of key Mendelian randomization findings for coagulation traits on acute subdural hematoma resolution. Horizontal bars indicate odds ratios (OR) for hematoma resolution with 95% reference line at OR = 1 (null). Red shading denotes protective association (fibrinogen γ'), while blue indicates harmful associations (factors VIII and XI). Numerical annotations display OR and corresponding *p* values. The schematic highlights that fibrinogen γ' increases the likelihood of hematoma clearance, whereas elevated factor VIII and XI activity reduce it.

Taken together, these MR findings are consistent with a plausible biological model in which coagulation dysfunction influences hematoma dynamics. In particular, a higher proportion of the γ' fibrinogen isoform may favor the breakdown and absorption of hematoma, whereas elevated activity of coagulation factors VIII and XI—which enhance thrombin generation and fibrin cross‐linking—likely contributes to the formation of denser fibrin clots that are more resistant to lysis, thereby impairing spontaneous hematoma resolution. Figure 8 (schematic placeholder) illustrates this concept: pro‐coagulant states yield dense, stable clots that tend to persist (and thus more often require surgical evacuation), whereas a mildly hypocoagulable or fibrinolysis‐favoring state (as conferred by higher fibrinogen γ' or anticoagulant proteins) could lead to faster clot degradation in vivo.

In the specific context of ASDH, hematoma clearance is thought to depend not only on systemic coagulation and fibrinolysis, but also on local processes within the subdural space, including inflammatory cell infiltration, organization of the subdural membranes, and drainage of liquefied blood products via arachnoid granulations. It is therefore plausible that coagulation profiles that promote a more easily lysable clot structure may facilitate these local clearance mechanisms, whereas highly stable fibrin networks may slow resorption and prolong inflammation. We emphasize that our MR results provide genetic evidence consistent with a causal influence of coagulation pathways on hematoma dynamics, but they do not by themselves prove the precise molecular or cellular mechanisms. The mechanistic interpretations proposed here should be regarded as hypotheses that require validation in dedicated experimental and clinical studies.

## 4 Discussion

In this two‐sample Mendelian randomization (MR) study, we provide genetic evidence consistent with a causal role of coagulation pathways in the spontaneous resolution of acute subdural hematoma (ASDH), as proxied by intracerebral hemorrhage (ICH) susceptibility. Our principal findings indicate that genetically higher fibrinogen γ' levels are associated with an increased likelihood of hematoma resolution, whereas elevated factor VIII and factor XI activity are associated with a reduced probability of spontaneous clearance. Other coagulation factors, natural anticoagulants, and platelet traits did not show significant associations with the hematoma‐related outcomes. These results were robust across multiple MR methods and sensitivity analyses [14]. A secondary analysis using 3‐month functional outcome after stroke as an alternative, more clinical proxy did not yield statistically significant effects, although the directions of the estimates were numerically similar. Given the limited power and indirect nature of this secondary outcome, we interpret those findings as exploratory and do not regard them as independent validation of the primary results.

### 4.1 Relation to Previous Studies

Our findings align with clinical observations that coagulation abnormalities influence outcomes after traumatic brain injury (TBI) and intracranial hemorrhage. Trauma‐induced coagulopathy has long been recognized as a determinant of hematoma expansion and mortality, yet its influence on hematoma resolution has been less well studied [7, 8, 9, 15, 16]. Prior observational cohorts have suggested that hypocoagulability or enhanced fibrinolysis may paradoxically facilitate clot breakdown and hematoma absorption [5, 6]. However, observational designs are inherently prone to confounding and reverse causation. By using genetic instruments as largely unconfounded proxies, our MR study strengthens the inference that specific coagulation traits—notably fibrinogen isoform composition and factor VIII/XI activity—are likely to play a causal role in the dynamics of hematoma resolution [10, 11, 12, 14, 17].

### 4.2 Biological Plausibility and Mechanisms

The biological mechanisms suggested by our results are broadly consistent with established hemostatic physiology. Fibrinogen γ', a splice variant of the fibrinogen γ chain, modifies fibrin clot structure and confers reduced cross‐linking, thereby rendering fibrin networks more susceptible to fibrinolysis [17, 18, 19, 20, 21]. A lifelong genetic predisposition to higher γ' fibrinogen levels may therefore increase the probability that intracranial hematomas form clots that are more amenable to lysis and subsequent clearance. Conversely, factors VIII and XI amplify thrombin generation and stabilize clot architecture, producing dense fibrin meshes that are relatively resistant to degradation. Excessive activity of these factors may hinder spontaneous clot resolution, leading to hematoma persistence and an increased need for surgical evacuation [18, 19, 22]. These interpretations are supported by experimental models of fibrin structure and by clinical studies linking elevated FVIII/FXI activity with thrombotic risk [18, 19, 22, 23].

In ASDH, however, hematoma resolution occurs within the subdural space rather than the brain parenchyma, and likely involves additional processes such as inflammatory cell infiltration, neomembrane formation, and drainage of liquefied blood products via arachnoid granulations. Coagulation factors may modulate these local processes indirectly by altering the physical properties and lysis susceptibility of the subdural clot [19, 21]. For example, a more porous, fibrinolysis‐prone clot structure could be cleared more efficiently by phagocytic cells and cerebrospinal fluid flow, whereas denser, highly cross‐linked fibrin may resist degradation and prolong local inflammation [18, 23]. We stress that our MR analysis cannot directly resolve these cellular mechanisms, and the path from genetic variation to subdural hematoma behavior remains inferential. Dedicated experimental and imaging studies will be needed to test these proposed mechanistic links in the specific context of traumatic ASDH.

### 4.3 Clinical Implications

From a translational standpoint, our findings highlight coagulation pathways as potential therapeutic targets to modulate hematoma dynamics in neurotrauma. The genetic evidence suggests that tailoring coagulation status—such as selectively attenuating FVIII/FXI activity or favoring a fibrinogen profile with higher γ' isoform content—might, in principle, facilitate hematoma absorption in patients with ASDH. While immediate clinical application is premature, these results provide a rationale for further exploration of individualized hemostatic strategies in traumatic brain injury.

In particular, several novel factor XI (FXI) inhibitors, including the small‐molecule FXIa inhibitor asundexian and the monoclonal anti‐FXI antibody abelacimab, are currently being evaluated in phase II–III clinical trials for thromboprophylaxis in non‐traumatic settings [24, 25, 26, 27, 28]. These agents have demonstrated the feasibility of selectively targeting FXI with relatively low rates of major bleeding, raising the possibility that FXI inhibition could be repurposed in carefully designed studies to investigate its impact on hemorrhage progression and resolution in trauma. Similarly, fibrinogen replacement therapies may need to consider the functional isoform balance, not just total fibrinogen concentration [20, 21, 29]. Any such translational strategies will, however, require rigorous safety evaluation and dedicated trials in patients with TBI and ASDH before they can be considered for clinical practice.

### 4.4 Strengths and Limitations

The strengths of this study include the use of large‐scale GWAS data for coagulation traits and hemorrhagic outcomes, a rigorous MR methodology, and extensive sensitivity analyses that reduce concerns about pleiotropy or weak instruments. We followed STROBE‐MR reporting standards to ensure transparency and reproducibility [13]. Nevertheless, several important limitations merit emphasis.

First, ASDH‐specific GWAS data on hematoma absorption or radiological resolution are not yet available; we therefore used ICH susceptibility and poststroke functional outcome as indirect proxies [11, 12, 14]. While these proxies are biologically related to intracranial bleeding and subsequent recovery, they cannot fully capture the unique pathophysiology and anatomical environment of traumatic ASDH. Spontaneous ICH typically occurs within the brain parenchyma and is often driven by hypertensive arteriopathy or cerebral amyloid angiopathy, whereas ASDH arises from traumatic tearing of bridging veins and evolves within the subdural space, where clearance depends on processes such as membrane formation and arachnoid granulation function. The extent to which the genetic architecture of ICH overlaps with the determinants of ASDH resolution is currently unknown. As such, our use of ICH and stroke outcomes as proxies should be viewed as a pragmatic but imperfect solution and may limit the direct translatability of our results to ASDH.

Second, the outcome datasets were of modest size, particularly for the 3‐month functional outcome GWAS, which limits statistical power and likely explains the absence of statistically significant findings in the secondary analysis. Third, our results are derived mainly from European ancestry populations, and their generalizability to other ethnic groups remains to be established [30]. Fourth, MR assumes lifelong genetic exposure, whereas the clinical course of ASDH is acute and time‐dependent; thus, the effect sizes estimated here may not directly map onto the impact of short‐term therapeutic interventions. These caveats underscore the need for cautious interpretation and for validation of our findings in ASDH‐specific clinical and imaging cohorts.

### 4.5 Future Directions

Future research should integrate prospective imaging–genetics cohorts of traumatic brain injury to directly validate these associations in patients with ASDH. High‐resolution CT or MRI markers of hematoma volume and absorption dynamics could serve as more specific outcomes in such studies. Parallel laboratory investigations into fibrinogen isoform biology and the impact of FVIII/FXI modulation on clot degradation in trauma settings are warranted. Ultimately, randomized controlled trials of targeted hemostatic interventions, guided by genetic or biomarker profiles, will be required to establish clinical efficacy and safety in this population [14, 24, 25, 26, 27, 28, 31].

## 5 Conclusion

This MR study provides genetic evidence that coagulation traits—particularly fibrinogen γ', factor VIII, and factor XI—are likely to exert causal influences on hematoma dynamics relevant to ASDH, as approximated by ICH susceptibility. By bridging genetic epidemiology with neurotrauma pathophysiology, our findings open new avenues for translational research and suggest that modulation of specific coagulation pathways may ultimately improve outcomes in patients with traumatic acute subdural hematoma. However, given the indirect nature of our outcome proxies and the differences between spontaneous ICH and traumatic ASDH, these results should be regarded as hypothesis‐generating and require confirmation in ASDH‐specific cohorts and mechanistic studies before informing clinical decision‐making.

## Author Contributions

Qizhong Wu, Tingting Xu, and Bo Tan conceived and designed the study. Qizhong Wu and Bo Tan performed data acquisition, analysis, and interpretation. All authors contributed to drafting and critically revising the manuscript for important intellectual content. All authors read and approved the final version of the manuscript. Qizhong Wu and Bo Tan contributed equally to this work and are co‐first authors.

## Funding

This research was supported by the following: Health Commission of Sichuan Province Medical Science and Technology Program (Grant No. 24WSXT042); Wu Jieping Medical Foundation (Grant No. 320.6750.2024‐6‐113); Guangyuan Science and Technology Bureau Science and Technology Project (Grant No. 23ZDYF0053); Sichuan Provincial Clinical Key Specialty Construction Project (Grant No. 2024HSWKP001).

## Ethics Statement

This study was conducted in accordance with the principles of the Declaration of Helsinki. No new human participants or animals were recruited, enrolled, or experimented upon. All data used were de‐identified summary‐level statistics from previously published genome‐wide association studies (GWAS), which are publicly accessible via open repositories. Therefore, institutional review board (IRB) approval and informed consent were not required.

## Consent

The authors have nothing to report. This study used only publicly available, de‐identified summary GWAS data and involved no individual‐level human data or identifiable personal information.

## Conflicts of Interest

The authors declare no conflicts of interest.

## Supporting information


**Table S1:** Summary of GWAS datasets used for coagulation traits in the Mendelian randomization analysis.

## Data Availability

Summary‐level GWAS data for coagulation‐related traits were obtained from the IEU OpenGWAS/MR‐Base database (https://gwas.mrcieu.ac.uk/). Outcome data were obtained from the International Stroke Genetics Consortium (ISGC), MEGASTROKE, and FinnGen, all of which are publicly accessible resources. All datasets used in this study are fully de‐identified and open‐access. The processed datasets and analysis scripts are available from the corresponding author upon reasonable request.
