# Supplementary material for: Coagulation Pathways as Determinants of Acute Subdural Hematoma Resolution: Genetic Evidence From Human Data
Source: CNS Neurosci Ther. 2026 Jan 7;32(1):e70741. doi: 10.1002/cns.70741 (PMC12778947; doi:10.1002/cns.70741)
Supplement: Supplementary file 1 — Table S1: Summary of GWAS datasets used for coagulation traits in the Mendelian randomization analysis. [file CNS-32-e70741-s001.docx]

Table S1 Summary of GWAS datasets used for coagulation traits in the Mendelian randomization analysis

| ****Exposure Trait**** | ****Sample Size**** | ****Ancestry/Population**** | ****Source Consortium/Publication**** |
| --- | --- | --- | --- |
| **Fibrinogen (Total)** | 50,000+ | European (predominantly) | IEU OpenGWAS, Fibrinogen GWAS Consortium |
| **Fibrinogen γ′** | 40,000+ | European (predominantly) | IEU OpenGWAS, Fibrinogen γ′ GWAS Consortium |
| **Factor V** | 100,000+ | European (predominantly) | MEGASTROKE, Coagulation Traits Consortium |
| **Factor VII** | 80,000+ | European (predominantly) | FinnGen, International Stroke Genetics Consortium |
| **Factor VIII** | 90,000+ | European (predominantly) | ISGC, MEGASTROKE Consortium |
| **Factor XI** | 85,000+ | European (predominantly) | MEGASTROKE, International Stroke Genetics Consortium |
| **Protein C** | 55,000+ | European (predominantly) | ISGC, Coagulation Factors GWAS Consortium |
| **Protein S** | 65,000+ | European (predominantly) | FinnGen, Coagulation Traits Consortium |
| **Antithrombin** | 50,000+ | European (predominantly) | ISGC, Coagulation Factors Consortium |
| **Platelet Count** | 70,000+ | European, Asian | MEGASTROKE, Platelet GWAS Consortium |
| **Platelet Volume** | 60,000+ | European (predominantly) | FinnGen, Platelet GWAS Consortium |

**Note:**
This table provides detailed information on all exposure GWAS datasets included in the Mendelian randomization analyses, including sample size, ancestry composition, and consortium/publication source. All datasets were derived from large-scale genome-wide association studies conducted predominantly in European populations. These details are provided in response to reviewer comments to enhance transparency, replicability, and assessment of instrument strength across exposure traits.
